# Epigenetic silencing of miR-19a-3p by cold atmospheric plasma contributes to proliferation inhibition of the MCF-7 breast cancer cell

**DOI:** 10.1038/srep30005

**Published:** 2016-07-21

**Authors:** Seungyeon Lee, Hyunkyung Lee, Hansol Bae, Eun H. Choi, Sun Jung Kim

**Affiliations:** 1 Department of Life Science, Dongguk University-Seoul, Goyang, Korea; 2 Plasma Bioscience Research Center, Kwangwoon University, Seoul, Korea

## Abstract

Cold atmospheric plasma (CAP) has been proposed as a useful cancer treatment option after showing higher induction of cell death in cancer cells than in normal cells. Although a few studies have contributed to elucidating the molecular mechanism by which CAP differentially inhibits cancer cell proliferation, no results are yet to be reported related to microRNA (miR). In this study, miR-19a-3p (miR-19a) was identified as a mediator of the cell proliferation-inhibitory effect of CAP in the MCF-7 breast cancer cell. CAP treatment of MCF-7 induced hypermethylation at the promoter CpG sites and downregulation of miR-19a, which was known as an oncomiR. The overexpression of miR-19a in MCF-7 increased cell proliferation, and CAP deteriorated the effect. The target genes of miR-19a, such as ABCA1 and PTEN, that had been suppressed by miR recovered their expression through CAP treatment. In addition, an inhibitor of reactive oxygen species that is produced by CAP suppressed the effect of CAP on cell proliferation. Taken together, the present study, to the best of authors’ knowledge, is the first to identify the involvement of a miR, which is dysregulated by the CAP and results in the anti-proliferation effect of CAP on cancer cells.

Cold atmospheric pressure plasma (CAP) is ionized media that mainly contains reactive oxygen species (ROS) and reactive nitrogen species (RNS)^1^. Ever since CAP was successfully produced in cold conditions, it has been given attention for medical applications, especially cancer treatment^2^. In fact, CAP has been widely proven to be able to differentially inhibit the growth of cancer cells compared with their normal counterpart in various cancer types. For example, when *in vitro*-cultured cancer cells, such as melanoma^3^, breast cancer^4^, and lung cancer cells^5^, were exposed to CAP, the cells showed growth retardation, increased double-strand breaks (DSB), and increased apoptosis. The CAP also worked effectively in *in vivo* animal models to treat xenografted cancer and promote wound healing. In detail, ovarian cancer cells xenografted into a mouse underwent cell death from the injection of CAP-activated medium^6^. In another study, CAP led to the successful skin wound healing of mice^7^. Clinically, patients suffering from chronic leg ulcers were treated in a clinically controlled monocentric trial with CAP^8^. The immediate antimicrobial effects of the CAP were almost comparable to octenidine treatment without any signs of cytotoxicity.

The molecular mechanism of how CAP changes cellular phenomena is being elucidated. So far, the main casts of CAP related to biological function are believed to be reactive nitrogen species (RNS) and reactive oxygen species (ROS)^9^. For ROS, its production and effect on cancer cells have been documented in many studies^10^,^11^,^12^. The ROS was shown to easily penetrate the cell membrane and diffuse to cytosol^13^, where it modulates or transduces diverse signaling, eventually leading to the regulation of gene expression. This phenomenon was supported in a series of studies that explained the same change of gene expression or cellular activity by CAP and ROS. An example is the observation of the dysregulation of TGF-α, VEGF^14^, and CDH1^15^ by either CAP or ROS. On the other hand, it seems that CAP also has unique regulatory pathways because in many cases CAP resulted in non-overlapping pathways with the reactive species. For example, in cervical cancer cells, the MAPK pathway was suppressed through decreasing MMP9 by CAP^16^. In another study, p53 and p21 were dysregulated by CAP^17^, which has not been observed in cells treated with reactive species.

CAP can induce a genetic change of DNA in the nucleus by producing a double-strand break (DSB). CAP-treated lung cancer cells showed DSB, thereby leading to apoptosis^5^. Whether CAP can directly induce DSB in the cell is yet to be elucidated, although it was shown to induce DSB in leucocytes embedded in agarose^18^. Other than DSB, little is known about genetic changes of DNA at a base level, such as the mutation of nucleotides. As an alternative explanation for the diverse changes of gene expression as well as cellular activities by CAP, epigenetic factors, such as CpG methylation, miR, and histone modification, have emerged^19^,^20^. Recently, in our previous study, a genome-wide methylation change by CAP in breast cancer cells was examined, and many cancer-related genes were revealed to have undergone methylation changes at their promoter CpG sites^21^.

In this study, we screened a collection of miRs to identify specific miRs that underwent a methylation change at the promoter CpG sites and thereby showed the alteration of gene expression by CAP. MiR-19a-3p (miR-19a) was found to have a good association between the methylation and expression level. To prove that miR-19a is truly regulated by CAP, the expression change for a few target genes of the miR were also examined. In addition, an ROS inhibitor was used to determine whether the effect of CAP on the expression of miR and target genes could be ablated.

## Results

### CAP induced hypermethylation and downregulation of miR-19a in MCF-7

**Figure 1:**
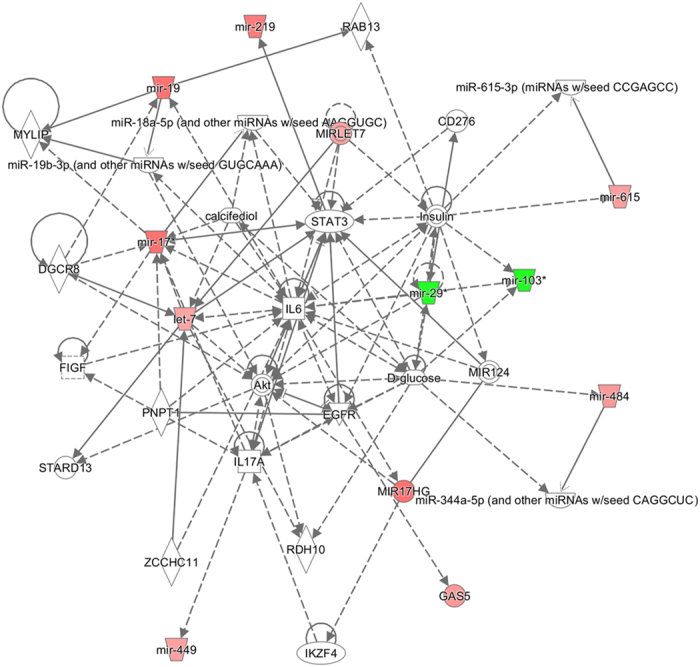
Top IPA network constructed by non-coding RNAs showing altered methylation by CAP in MCF-7. The highest confidence network was constructed using IPA with 43 dysregulated CpGs of non-coding RNAs. The top network was “Cancer, Organismal Injury and Abnormalities, Gastrointestinal Disease.” Hypermethylated genes are shaded in red, while hypomethylated genes are shaded in green, with the color intensity signifying the magnitude of methylation change. Solid and dashed lines represent direct and indirect interactions, respectively.

**Figure 2:**
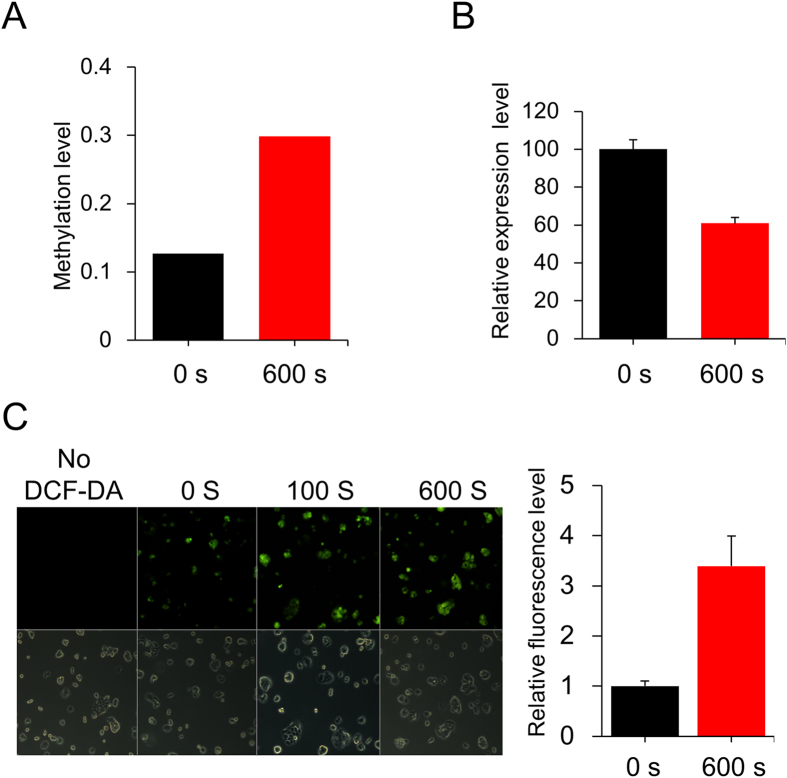
Induction of hypermethylation and downregulation of miR-19a in MCF-7 by CAP. (**A**) Methylation level of CpGs at the promoter of miR-19a after CAP treatment. The data are from methylation microarray analysis of MCF-7 treated with CAP. (**B**) Real-time RT-PCR analysis of miR-19a (Mean ± SE of three replicates). (**C**) Fluorescence microscopic images of the MCF-7 cell after treating the cell with CAP for 0, 100, and 600 s following addition of DCF-DA. The bottom panel shows images from bright-field microscopy. The bar graph to the right shows quantification of fluorescence intensity for 600 s (Mean ± SE of three replicates).

In a previous study, we carried out a genome-wide methylation chip analysis and identified a group of genes for which the methylation status was significantly altered by CAP^21^. In the list of affected genes, also included were the promoter CpG sites of miRs. The array covered 42 noncoding RNAs, including 17 miRs (Table 1), and 25 long noncoding RNAs (Supplementary Table S1) showed a significant methylation change, satisfying our screening criteria (β-values equal to or greater than 0.15). An Ingenuity pathway analysis with the 42 non-coding RNAs identified “Cancer, Organismal Injury and Abnormalities, Gastrointestinal Disease” as the top interaction network (Fig. 1). Notably, a few miRs, including miR-19a, which have been known to be involved in carcinogenesis showed altered methylation by CAP (Fig. 2A). For example, miR-484 is known as an oncomir in breast cancer^22^, which is hypermethylated by CAP (Δβ = 0.153). Additionally, miR-17, which is known as an oncomir in hepatocellular carcinoma^23^, is hypermethylated (Δβ = 0.172). Meanwhile, miR-29 (Δβ = −0.174) and miR-103 (Δβ = −0.187), which have tumor-suppressive activity in prostate cancer^24^,^25^, are hypomethylated.

In further studies, we paid attention to the oncogenic miRs (called oncomiRs). We hypothesized that the hypermethylation of the oncomirs by CAP would accompany the downregulation of gene expression, eventually resulting in a decrease of cell proliferation. Among the 17 miRs, miR-19a, miR-17^23^, and miR-18a^26^ were identified as oncomiRs from the PubMed database search. RT-PCR analysis revealed that only miR-19a showed a significant downregulation of expression, with an approximately 40% decrease by CAP treatment (Fig. 2B,C). MiR-19a, therefore, was selected to further examine its role in mediating the effect of CAP on the inhibition of cancer cell proliferation.

### CAP deteriorated cell-proliferative effect of miR-19a in MCF-7

**Figure 3:**
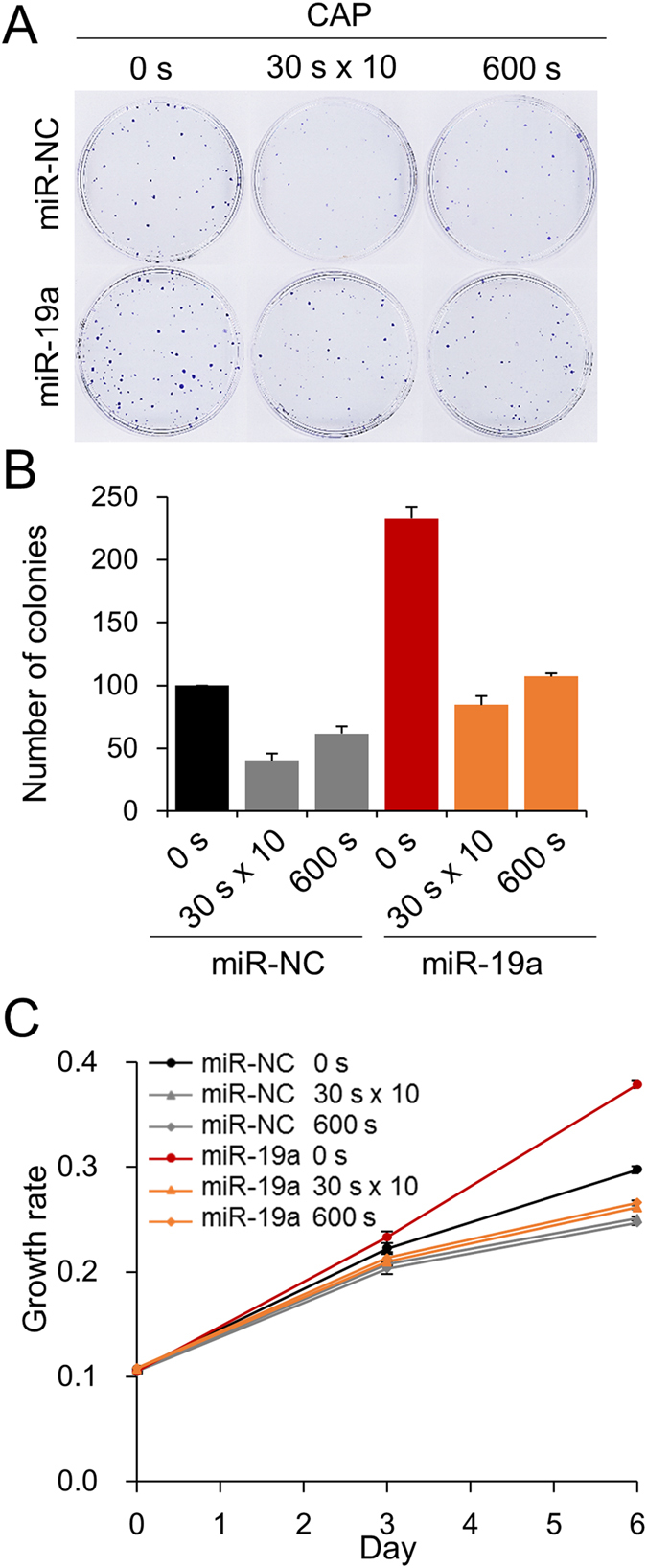
CAP suppresses cell proliferation driven by miR-19a. The effect of CAP on miR-19a-transfected MCF-7 was examined by colony formation assay (**A**). Transfected cells were treated with CAP by 600 s or 10 times for 30 sec scheme. The number of colonies was counted using ImageJ^47^ and indicated as a bar graph (**B**). The effect of CAP was also analyzed by dye-based cell proliferation assay (**C**). MiR-transfected cells were treated with CAP and assayed at day 0, 3, and 6. MiR-NC; control miR. All the assays were performed in triplicates, and the result is depicted as Mean ± SE.

MiR-19a has been known to stimulate cell proliferation in a few cancers, including gastric and pancreatic cancer, where the miR is upregulated, leading to poor prognosis in patients^27^,^28^. We also found pro-proliferation activity in the miR-19a-overexpressing MCF-7 cell. When the miR-19a mimic was transfected into the cell, the size as well as the number of cell colonies were increased 2.5-fold compared with the control miR (Fig. 3A,B). When CAP was used to treat the miR-19a-overexpressing cell, it induced a decrease of the area occupied by the colonies. The two treatment conditions of CAP, 10 times for 30 sec and a single dose for 600 sec, had a similar effect of proliferation inhibition with the former condition, showing a slightly stronger effect. The effect of CAP on cell proliferation was monitored by another method by examining the growth rate of the cells using a dye-based analysis. The result indicated that both conditions of CAP retarded the growth of the MCF-7 cell (Fig. 3C). Taken together, miR-19a stimulates cell proliferation and CAP is able to deteriorate miR’s effect.

### Target genes of miR-19a were upregulated by CAP in MCF-7

**Figure 4:**
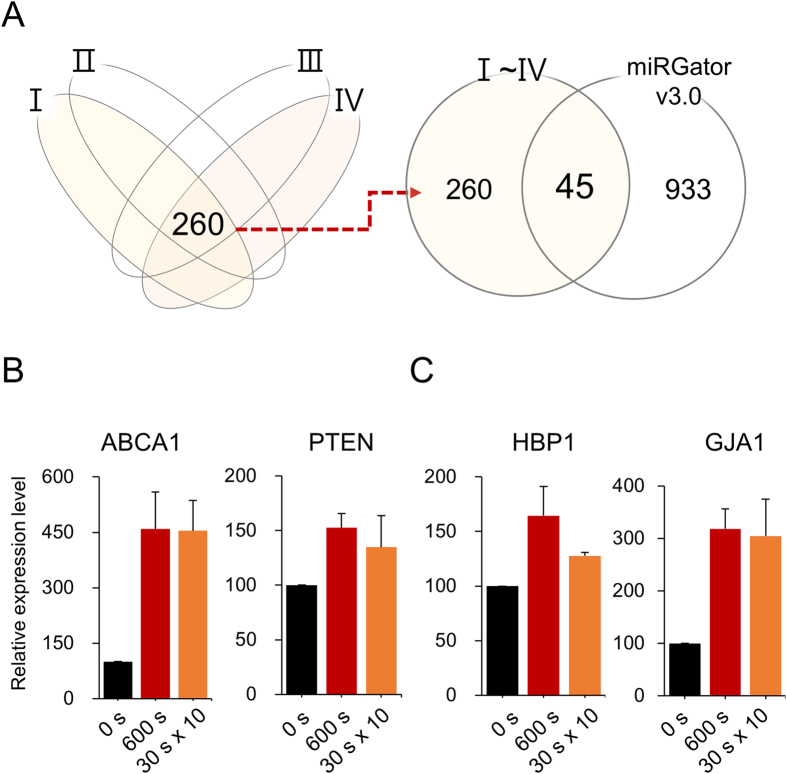
Upregulation of target genes of miR-19a by CAP treatment of MCF-7. (**A**) The strategy for selection of target genes of miR-19a. Potential targets were initially identified using four target gene prediction programs (miRWalk, miRanda, TargetScan, miRDB [I-IV]), and the resulting 260 genes were compared with miRGator to finally obtain 45 genes. (**B**) Upregulation of ABCA1 and PTEN, which were previously known as the target genes of miR-19a. (**C**) Upregulation of HBP1 and GJA1, which were newly identified as targets of miR-19a in this study. All the experiments were performed in triplicates, and the result is depicted as Mean ± SE.

**Figure 5:**
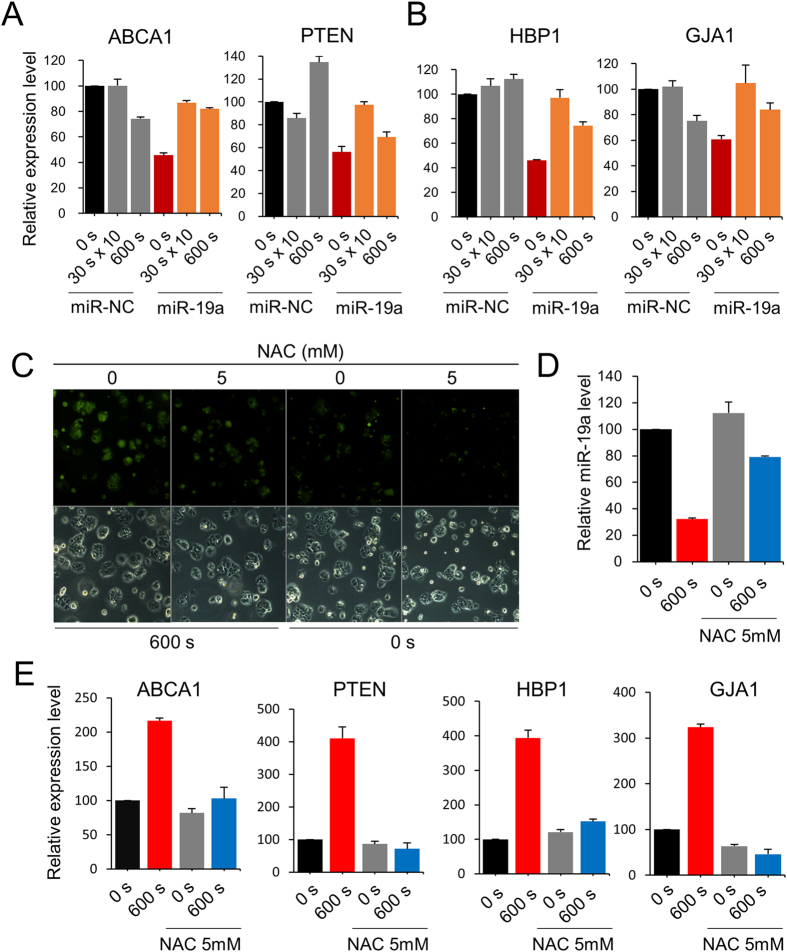
CAP deteriorates the downregulation effect of miR-19a on target genes. After transfection of mimic or control (NC) miR into MCF-7, the cells were treated with CAP for 600 s or 10 times for 30 s. Expression of indicated genes was examined by quantitative RT-PCR. (**A**) Expression of ABCA1 and PTEN, which were previously known as target genes of miR-19a. (**B**) Expression of HBP1 and GJA1, which were newly identified as target genes of miR-19a in this study. (**C**) Fluorescence microscopic images of the MCF-7 cell after treating the cell with an ROS inhibitor, N-Acetyl-L-cysteine. The bottom panel shows images from bright-field microscopy. Effect of ROS inhibitor on the expression of miR-19a (**D**) and target genes (**E**) were analyzed by quantitative RT-PCR. All the experiments were performed in triplicates, and the result is depicted as Mean ± SE.

As miRs manifest their activity through binding and inactivating target RNAs, miR-19a’s targets were first identified using four target gene prediction programs (miRWalk, miRanda, TargetScan, and miRDB) and a deep sequencing database, miRGator v3.0 ( http://mirgator.kobic.re.kr)^29^, which resulted in 45 genes as potential targets (Fig. 4A). Among them, four genes (ABCA1, PTEN, HBP1, and GJA1) were randomly selected to examine their expression change by CAP treatment. ABCA1^30^ and PTEN^31^ have already been known as miR-19a’s target, but the other two are yet to be experimentally determined. As shown in Fig. 4B,C, all four genes were upregulated by the CAP treatment, explaining the intimate association of expression between miR-19a and its target genes. Next, the expression of target genes was examined in cells that were transfected with miR-19a mimic and then treated with CAP. In all four genes, the expression was downregulated by the overexpressed miR-19a; however, it was restored by CAP treatment (Fig. 5A,B). This indicates the recovery of the target gene activity by CAP through the inhibition of miR-19a, which had been suppressed by the miR.

It has been suggested that the molecular and cellular events provoked by CAP are mediated mainly by ROS. To examine whether the expression change of the miR as well as its target genes occurs in this scenario, their expression is monitored after CAP treatment in the presence of an ROS inhibitor. As shown in Fig. 5C, the ROS level was decreased by an inhibitor, N-Acetyl-L-cysteine (NAC). The expression of miR-19a was less alleviated (Fig. 5D); however, the expression of target genes was suppressed by NAC even after treatment of CAP (Fig. 5E), suggesting the regulation of miR-19a by ROS.

## Discussion

This study was carried out to identify miRs, especially oncogenic ones (oncomiR), which drive cells to be cancerous and are suppressed by CAP, for the purpose of elucidating the epigenetic mechanism of CAP’s anti-cancer effect. Currently, the molecular mechanism for how CAP affects the expression of genes is unclear. When CAP is used to treat the cell culture medium, its effect is transduced to the cytoplasm or nucleus via ROS and RNS (RONS)^32^. The RONS then acts on proteins, provoking changes of a variety of cellular activities, such as growth inhibition, apoptosis, and necrotic cell death^33^,^34^. In many cases, these changes are caused by the alteration of gene expression. For example, the activation of the MAPK and p53 signaling pathways was found as a result of CAP exposure in the non-small-cell lung cancer A549 cell, resulting in changes of expression for MEKK, GADD, FOS, and JUN^35^. The MAPK signaling pathways seem to be a major CAP target because they appeared in other cell types, including immune cell lines (Jurkat and THP-1)^36^ and melanoma cells^37^. In another study, CAP-induced ROS disturbed the mitochondrial-nuclear network in the A549 lung cancer cell through a caspase-independent apoptotic pathway by downregulating the anti-apoptotic protein Bcl2, but by no activation of caspase 3/7^32^.

Another possible explanation is the regulation of methylation status at the CpG sites of the promoter, as shown for the miR-19a in this study. In our previous study, CAP was also able to change the methylation status at the promoter CpG sites of a set of coding genes that were involved in cell proliferation^21^. Further future studies should focus on elucidating how CAP regulates factors, mainly methyltransferase, to adjust the methylation level.

It is impressive that CAP suppressed the pro-proliferative effect of the oncomiR by upregulating its target genes, such as ABCA1, PTEN, HBP1, and GJA1, when CAP was used to treat the miR-19a-transfected cell. Interestingly, ABCA1 and PTEN are targets of TGF-β, and their downregulation is associated with poor prognosis in various cancers, including ovarian^38^ and breast cancer^39^,^40^. HBP1 is a tumor suppressor whose expression is lower in breast tumors targeted by the PIα/FOXO pathway^41^. GJA1 (also known as Connexin-43) is a connexin family protein and forms the gap junction. It is expressed primarily in myoepithelial cells and inhibits mammary gland tumor metastasis^42^. These characteristics of target genes as tumor suppressors in various cancer types support the suppressive effect of CAP against miR-19a.

A wide range of CAP treatment times have been applied to cells and successfully resulted in cell death. The treatment of head and neck squamous cancer cells with a surface micro-discharging (SMD) CAP for 30 s–180 s decreased the cell viability in all treatment times^43^. When Hattori *et al*. pre-treated the culture medium for 1–5 min and added it to pancreatic cancer cells, the cell growth of all the treated groups was inhibited^44^.

In this study, two different strength conditions of CAP were applied to cells: a single shot of 600 s and 10 times for 30 s with a one-hour interval. Both the adopted strength ranges of CAP have shown the same cellular phenomena in terms of cell proliferation and apoptosis, although the 10 times for 30 s shot showed stronger cell proliferation inhibition. Furthermore, the target genes also showed higher expression changes in the case of the 10 times for 30 s shot. So far, a few signals of DBD CAP with different powers and duration times have been successfully applied to cancer cells to induce cell death with a specific gene expression change. Our study and previous studies suggest the possibility of designing a specific CAP strength that can modulate specific cellular activity and gene expression.

Previous studies have shown a higher rate of cell death in a variety of cancer cells than in normal cells, such as lung and melanoma cancer^5^,^45^,^46^. This phenomenon is believed to be at least in part due to the elevated level of ROS in cancer cells, and CAP accumulates more ROS upon it, eventually leading to cell death^45^. We also found downregulation of miR-19a in MDA-MB-231 other than MCF-7 (Supplementary Fig. S1), supporting the same effect of CAP on the downregulation of miR-19a in different cell types. However, more cell types should be examined to determine whether the downregulation of the miR in cancer is a general observation. It should also be mentioned that the molecular mechanism for how the signal from CAP is transduced to the alteration of miR-19a expression has yet to be elucidated. The alteration of the CpG methylation at the miR’s promoter could give insight into identifying mediators in the CAP-elicited deregulation of miR-19a and its target genes.

In conclusion, this study identified that miR-19a, known as an oncomiR, underwent hypermethylation and downregulation and that the target genes of miR-19a were upregulated in the MCF-7 breast cancer cell by CAP treatment. In addition, the cell proliferation effect after the overexpression of miR-19a was suppressed by CAP. These results could contribute to establishing the epigenetic mechanism of CAP when it is applied to cells as well as tissues for cancer treatment application.

## Materials and Methods

### Cell culture and cold atmospheric CAP treatment of cells

The human breast cancer cell lines MCF-7 and MDA-MB-231 were purchased from the American Type Culture Collection (ATCC) (Manassas, VA, USA) and cultured in RPMI 1640 (Gibco, Los Angeles, USA) supplemented with 10% FBS (Gibco) and 2% Penicillin and Streptomycin (Gibco) under humid conditions with 5% CO_2_ at 37 °C. The electrode type of the CAP device, manufactured at the Plasma Bioscience Research Center (Kwangwoon University, Korea), was mesh-DBD (Dielectric Barrier Discharge). The discharged voltage was measured at a strength of 0.46 kV when the supply voltage was set to 0.12 kV with 1.5 L/min of argon gas as a feeding gas. The chosen operational frequency was 12.89 kHz. Cells in the culture media were exposed to the CAP source ten times for 30 seconds every hour or one time for 100 sec or 600 sec.

### MicroRNA transfection

MiR-19a mimic and miR-negative control (miR-NC) were synthesized by Bioneer (Korea) (Supplementary Table S2), and their sequences were based on the miRBase sequence database ( http://www.mirbase.org/). To perform the overexpression study of miR-19a, the miR and its negative control were transiently transfected into the cells in 50–60% confluency at 30 nM of the final concentration using Lipofectamine 2000 reagent (Invitrogen, Carlsbad, CA, USA) in serum-free Opti-MEM I Medium (Gibco). Twenty-four hours after the transfection, the cells were harvested for RNA isolation.

### RNA extraction and Real-time RT-PCR

Total RNA was extracted at least twenty-four hours after CAP treatment from cells using the ZR-Duet DNA/RNA MiniPrep kit (Zymo Research, Irvine, CA, USA) following the manufacturer’s protocol. To quantify the level of mature miR-19a, reverse transcription with the extracted RNA was carried out using the miScript II RT Kit (Qiagen, Valencia, CA, USA). Quantitative RT-PCR was then conducted with the miScript SYBR Green PCR Kit (Qiagen) and miScript Primer Assays as the primers. In the case of the quantification of protein-coding genes’ expression level, cDNA was synthesized using ReverTra Ace qPCR RT Master Mix with gDNA remover (Toyobo, Japan), and KAPA SYBR FAST qPCR Kit Master Mix ABI Prism (Kapa Biosystems, Inc., Wilmington, MA, USA) was used for real-time PCR reaction. U6 and GAPDH were used for normalizing the expression of miR-19a and the protein-coding genes, respectively, with the 2^−ΔΔCt^ method. Each RT-PCR reaction was assayed in triplicate on an ABI 7300 instrument (Applied Biosystems, Foster City, CA, USA). The primers used for the amplification of miRs and selected genes are listed in Supplementary Table S2.

### Cell proliferation assay

Cell growth was monitored using CCK-8 assay (Dojindo Molecular Technologies, MD, USA), which is a colorimetric assay using water-soluble tetrazolium salts that generate a yellow or orange formazan dye through interaction with viable cells. 3 × 10^3^ cells/well of MCF-7 were seeded in 96-well plates in triplicate. Twenty-four hours after seeding, 30 nM of miR-19a mimic and control miR were transiently transfected using Lipofectamine 2000 reagent (Invitrogen) and cultured at 0, 3, and 6 days at 37 °C in a humidified environment of 5% CO_2_. One-tenth of the media volume of CCK-8 reagent solution was then added into each well and incubated for two hours. The absorbance value at 450 nm (A450) was measured with a microplate reader, as was the absorbance at 600 nm (A600) as a reference. The effect of turbidity was eliminated by subtracting the A600 value from the A450 value of the same well.

### Colony formation assay

5 × 10^3^ MCF-7 cells transfected with miR mimic or control miR in a 60-mm culture dish were maintained in a humidified atmosphere of 5% CO_2_ at 37 °C for 14 days. After being fixed (methanol:acetic acid = 7:1), the colonies were stained with 0.5% crystal violet for one hour at room temperature and washed with 1xPBS (Gibco). The colonies were counted using ImageJ software (National Institutes of Health, Bethesda, MD, USA).

### Extraction of differentially methylated miRs by CAP

Differentially methylated CpGs in the promoter of the non-coding RNA were extracted from previously published Illumina Infinium Human Methylation 450 K array data from NCBI’s GEO dataset (GEO accession number: GSE65085) with the criteria of |Δβ| ≥ 0.15 after removing the observations with a *p*-value of ≥0.05. Δβ was calculated by subtracting the methylation level (β value) of the non-treated cells from that of the CAP-treated cells.

### Measurement of reactive oxygen species levels (ROS)

The generation of ROS in cultured cells treated with CAP was quantified using the cell-permeant fluorescence probe, 2′, 7′-dichlorofluorescin diacetate (DCFH-DA; Sigma-Aldrich, St Louis, MO, USA). Cells were stained with 20 μM of DCFH-DA for 30 min at 37 °C and then exposed to CAP for 0, 100, and 600 sec. Thirty minutes after incubation at 37 °C in the dark, the fluorescence intensities of the DCFH-DA in the cells displaying intracellular ROS levels were detected by fluorescence microscopy (Leica Microsystems, Germany) and quantified using Infinite 200 Pro fluorescence reader (Tecan, Switzerland). To inhibit the production of intracellular ROS, 5 mM of N-Acetyl-L-cysteine (NAC; Sigma-Aldrich), known as a scavenger of ROS, was used to treat 5 × 10^5^ cells in a 60-mm culture dish. After incubation for two hours, the cells were exposed to CAP and the intracellular ROS levels were measured with the aforementioned procedure.

### Statistical analysis

All experimental results were plotted using Microsoft Excel software (2013 for Windows) as the mean ± standard error of three independent experiments. Student’s t-test was carried out for checking the statistical significance. Differences were considered statistically significant when the *p*-value was lower than 0.05.

## Additional Information

**How to cite this article**: Lee, S. *et al*. Epigenetic silencing of miR-19a-3p by cold atmospheric plasma contributes to proliferation inhibition of the MCF-7 breast cancer cell. *Sci. Rep.* **6**, 30005; doi: 10.1038/srep30005 (2016).

## Supplementary Material

Supplementary Information

## Figures and Tables

**Table 1:** MicroRNAs displaying differential methylation in MCF-7 cells exposed to CAP.

**Symbol**	**Accession no.**	**Description**	**Delta mean**	**Fold change**	**Target ID**
MIR219A1	NR_029633	microRNA 219a-1	0.1854	2.1735	cg14363494
MIR2117	NR_031751	microRNA 2117	0.1842	2.1397	cg03184776
MIR449C	NR_031572	microRNA 449c	0.1750	1.5578	cg14079131
MIR18A	NR_029488	microRNA 18a	0.1717	2.3564	cg07235355
MIR17	NR_029487	microRNA 17	0.1717	2.3564	cg07235355
MIR19A	NR_029489	miRNA 19a	0.1717	2.3564	cg07235355
MIR4258	NR_036212	microRNA 4258	0.1687	1.9979	cg06418219
MIR5692A1	NR_049875	microRNA 5692a-1	0.1667	2.6457	cg03068446
MIR615	NR_030753	microRNA 615	0.1570	1.6089	cg14644523
MIR484	NR_030159	microRNA 484	0.1536	1.6919	cg06397424
MIR3143	NR_036096	microRNA 3143	0.1532	1.6837	cg21201844
MIRLET7I	NR_029661	microRNA let-7i	0.1528	1.5182	cg06162516
MIR5189	NR_049821	microRNA 5189	−0.1575	−1.2741	cg02383666
MIR29C	NR_029832	miRNA 29c	−0.1737	−1.2691	cg00823526
MIR29B2*	NR_029518	microRNA 29b-2	−0.1737	−1.2691	cg00823526
			−0.1872	−1.2709	cg22525895
MIR103A2	NR_029519	microRNA 103a-2	−0.1865	−1.3055	cg12617066
MIR103B2	NR_031722	microRNA 103b-2	−0.1865	−1.3055	cg12617066

^*^MIR29B2 contains two CpG sites with differential methylation.
