# Supplementary material for: Epigenetic silencing of miR-19a-3p by cold atmospheric plasma contributes to proliferation inhibition of the MCF-7 breast cancer cell
Source: Sci Rep. 2016 Jul 21;6:30005. doi: 10.1038/srep30005 (PMC4956745; doi:10.1038/srep30005)

Epigenetic silencing of miR-19a-3p by cold atmospheric plasma contributes to proliferation inhibition of the MCF-7 breast cancer cell

Seungyeon Lee, Hyunkyung Lee, Hansol Bae, Eun H. Choi, and Sun Jung Kim

Supporting Information

Table S1. Long noncoding RNAs displaying differential methylation in MCF-7 cells exposed to CAP

Symbol	Accession No.	Description	Δ β-value	Fold change	Target ID
PRR7-AS1	NR_038915	PRR7 antisense RNA 1	0.5003	3.4100	cg06189653
SNHG5	NR_003038	Small nucleolar RNA host gene 5	0.2488	2.8294	cg17490806
NOP14-AS1	NR_015453	NOP14 antisense RNA 1	0.2324	2.4779	cg01190037
ST7-AS1	NR_002330	ST7 antisense RNA 1	0.2186	2.6955	cg08899199
CAHM	NR_037593	Colon adenocarcinoma hypermethylated	0.2001	1.8242	cg25157874
MIR100HG	NR_024430	Mir-100-let-7a-2 cluster host gene	0.1998	1.7177	cg03261272
ZNRD1-AS1	NR_026751	Zinc ribbon domain containing 1 antisense	0.1989	2.1526	cg02078039
LSAMP-AS3	NR_015391	LSAMP antisense RNA 3	0.1854	1.7804	cg09237790
FTX	NR_028379	FTX transcript, XIST regulator	0.1820	1.4135	cg04667267
SNHG11	NR_003239	Small nucleolar RNA host gene 11	0.1770	2.0084	cg17526424
KTN1-AS1	NR_027123	KTN1 antisense RNA 1	0.1719	2.5794	cg24579896
MIR17HG	NR_027349	MiR-17-92a-1 cluster host gene	0.1717	2.3564	cg07235355
ZNFX1-AS1	NR_003604	ZNFX1 antisense RNA 1	0.1625	1.7721	cg00759619
MESTIT1	NR_004382	MEST intronic transcript 1, antisense RNA	0.1619	1.6647	cg22115706
GAS5	NR_002578	Growth arrest specific 5	0.1609	1.6991	cg07177756
THAP7-AS1	NR_027051	THAP7 antisense RNA 1	0.1608	2.0341	cg17353431
IPW	NR_023915	Imprinted in Prader-Willi syndrome	0.1605	1.5740	cg11811341
FAM66D	NR_027425	Family with sequence similarity 66 member D	0.1568	1.2414	cg07467338
ZNF674-AS1	NR_015378	ZNF674 antisense RNA 1	0.1567	1.4621	cg01166930
MKNK1-AS1	NR_038403	MKNK1 antisense RNA 1	0.1566	1.2298	cg07228905
LOC400027	NR_028408	Long intergenic non-protein coding RNA 938	0.1553	2.2106	cg00035453
LBX2-AS1	NR_024606	LBX2 antisense RNA 1	0.1547	1.3178	cg03270710
SOCS2-AS1	NR_038263	SOCS2 antisense RNA 1	0.1532	1.6106	cg17051560
LINC00461	NR_024384	Long intergenic non-protein coding RNA 461	0.1526	1.6861	cg04270358
XIST	NR_001564	X inactive specific transcript	-0.1664	-1.2908	cg17513789

Table S2. Information of microRNA mimics and primers employed in this study

	MiR or Gene	Catalog no.	Product name or sequence (5'-3')	Supplier
**MiR**				
mimic	miR-19a-3p		UGUGCAAAUCUAUGCAAAACUGA	Bioneer
control	control miR	SMC-3001	miRNA mimic Negative control #2	Bioneer
**Primer**	miR-19a-3p	MS00003192	Hs_miR-19a_1 miScript Primer Assay	Qiagen
	RNU6	MS00033740	Hs_RNU6-2_11 miScript Primer Assay	Qiagen
	ABCA1		F: ACGACCACCATGTCAATCCT	Genotech
			R: TCGACAGTCAGCATGTCAAA	
	PTEN		F: TGTGCCTGTTTGACCTCTGA	Genotech
			R: CACAAACTGAGGATTGCAAGTT	
	HBP1		F: TTTTGAAAGGCACACGACTG	Genotech
			R: ACCATAGCCCTTGTGAATGC	
	GJA1		F: GACAGGTCTGAGTGCCTGAA	Genotech
			R: GGGCACCACTCTTTTGCTTA	
	GAPDH		F: ACATCGCTCAGACACCATG	IDT
			R: TGTAGTTGAGGTCAATGAAGGG	

Figure S1. Downregulation of miR-19a in the MDA-MB-231 cell by CAP. MDA-MB-231 cells were treated with CAP for 600 s and further cultured for 24 h. The expression of miR-19a was examined by real-time RT-PCR analysis (Mean ± SE of three replicates). The cells not treated with CAP (0 s) were used as control.

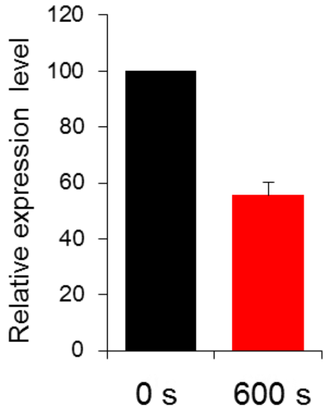

Supplement: Supplementary Information [file srep30005-s1.pdf]
